# Randomised clinical trial of extended depth of focus lenses for controlling myopia progression: Outcomes from SEED LVPEI Indian Myopia Study

**DOI:** 10.1136/bjo-2023-323651

**Published:** 2024-04-11

**Authors:** Manoj K Manoharan, Pavan K Verkicharla

**Affiliations:** 1 Infor Myopia Centre, L V Prasad Eye Institute, Hyderabad, Telangana, India; 2 Myopia Research Lab, Brien Holden Institute of Optometry and Vision Sciences, Prof. Brien Holden Eye Research Centre, L V Prasad Eye Institute, Hyderabad, Telangana, India

**Keywords:** Vision, Clinical Trial, Contact lens

## Abstract

**Purpose:**

To determine the efficacy of extended depth of focus (EDOF) contact lenses for controlling myopia progression in children through a 1-year randomised clinical trial.

**Methods:**

A total of 104 children aged 7–15 years, with spherical equivalent refraction ≤−0.50 D, were randomly assigned to wear SEED 1 dayPure EDOF Mid contact lenses (n=48) or single vision spectacle lenses (n=56). Cycloplegic refraction with Shin-Nippon open field autorefractor and axial length with Lenstar LS 900 was determined at the baseline and 12-month visits. The compliance, visual discomfort and dryness questionnaires were administered during the final visit.

**Results:**

Sixty-nine children (control: n=38; treatment: 31) completed the 12-month follow-up visit, with no difference in baseline characteristics between the groups. Mean (SEM) myopia progression in the 12th month was −0.48±0.07D in the control group and −0.20±0.08D in the treatment group. Mean axial elongation was 0.22±0.03 mm and 0.11±0.03 mm in the control and treatment groups, respectively. SEED 1 dayPure EDOF Mid contact lenses slowed myopia progression by 59% (−0.28D; p=0.01) based on spherical equivalent refraction and controlled axial length by 49% (0.11 mm; p=0.007) in comparison to single vision spectacle lenses. None of the participants reported any adverse effects. While most of the participants (82%) were comfortable with the contact lenses, 11% reported occasional dryness and 14% experienced mild fluctuations in visual acuity after immediate lens wear.

**Conclusion:**

Daily wear of SEED 1 dayPure EDOF Mid contact lenses in Indian children showed a significant effect in controlling myopia progression and axial elongation.

**WHAT IS ALREADY KNOWN ON THIS TOPIC**

- Contact lenses based on an extended depth of focus (EDOF) design were reported to slow myopia progression and axial elongation in children of Chinese ethnicity.
- However, it is not clear if these EDOF-design contact lenses have similar myopia control effects in Indian children, who have been reported to have a lower magnitude of peripheral defocus than children of other ethnicities.

**WHAT THIS STUDY ADDS**

- SEED 1 dayPure EDOF Mid contact lenses up to +1.50D showed a significant reduction in myopia progression and axial length elongation compared with that of the conventional single-vision spectacles in Indian children.

**HOW THIS STUDY MIGHT AFFECT RESEARCH, PRACTICE OR POLICY**

- These contact lenses can play a role in controlling myopia progression and axial elongation, which inturn may help in preventing visually debilitating complications associated with myopia progression and high myopia.

## Introduction

Myopia appears to be a highly prevalent refractive condition in multiple countries and is considered a global health problem of the 21st century.^1 2^ The increasing prevalence and the progression of myopia worldwide led to the development of various myopia control strategies for progression.^3^ Previous studies reported that the conventional correction of myopia using single-vision spectacle lenses increases the hyperopic defocus in the retinal periphery.^4 5^ Considering the potential role of peripheral retinal signals in myopia and to counteract the relative peripheral hyperopic defocus,^6–8^ peripheral defocus spectacles^9 10^ and contact lenses^11–13^ (multifocal or aspheric optics) were developed to induce myopic defocus in the peripheral retina to control the axial elongation.

Previous studies indicated that the design of the^1^ Defocus Incorporated Soft Contact (DISC)^11^ lenses with concentric alternate distance and defocus zones (myopic defocus) that cover the pupil and the^2^ Dual-Focus optical design lenses^12^ with centre-distance optics slowed the myopia progression compared with that of single vision contact lenses. More recently, the extended depth of focus (EDOF) design lenses were investigated for their efficacy in myopia control.^14 15^ Bakaraju *et al* 16 investigated the optical performance of EDOF design lenses and reported improvement in retinal image quality with these lenses compared with that of other multifocal contact lenses for intermediate and near viewing without compromising the distance vision.^17^ Sankaridurg *et al* investigated EDOF lenses (+1.25D and +1.75D) that were designed to alter global retinal image quality by improving the points on and in front of the retina while degrading the points behind the retina and reported that these lenses slow myopia progression (24%–32%) and axial elongation (22%–32%) significantly in Chinese ethnicity.^14^

The efficacy of these EDOF design lenses was, however, not investigated in children of other ethnicities, including Indian children. We hypothesise that the treatment effect will be different in Indian children compared with Chinese children as a recent study reported that Indian children have a relatively lower magnitude of peripheral defocus^18^ than other ethnicities,^19–21^ with an asymmetrical peripheral refraction pattern. The purpose of this study was to determine the efficacy of SEED 1 dayPure EDOF up to +1.50D (mid) contact lens in controlling myopia progression and axial elongation. In addition, we investigated comfort and visual experience with the SEED’s EDOF contact lenses through a questionnaire.

## Materials and methods

### Study design

This is a prospective, randomised and parallel-arm clinical trial conducted at LV Prasad Eye Institute (LVPEI), Hyderabad, India from December 2020 to August 2022. Written informed consent and written assent were obtained from the parent/guardian and child, respectively, post the explanation of the study and experimental protocol. This study was registered in ClinicalTrials.gov (Identifier number: NCT04618510) and Clinical Trials Registry—India (CTRI/2021/01/030839).

### Participants

The recruitment of research participants was conducted through public advertisements and via phone calls based on information of children from the electronic medical record database of LVPEI. Participants were randomly allocated to wear either SEED 1 dayPure EDOF Mid (+1.50D) contact lenses or single-vision spectacle lenses in a 1:1 ratio. The sequence of allocation was generated through Microsoft Excel 2016. Neither the participants nor the investigator was masked during the study duration.

#### Inclusion criteria

- Myopia (spherical equivalent refraction, SER) between −0.50D and −10.00D.
- Astigmatism <0.75D.
- Anisometropia <1.00D.
- Age ranging from 7 to 15 years.
- Neophyte/existing soft contact lens wearer.
- Best-corrected visual acuity: ≥20/20.
- Participants who are willing to wear contact lenses every day during the study period.

#### Exclusion criteria

- Any ocular or systemic conditions that could influence the refractive error.
- Current/prior use of orthokeratology lenses/bifocals/myopia control intervention.
- Use of any medications or eye-drops that could influence the refractive error.

### Intervention and control

Individuals with myopia in the control group wore single-vision spectacle lenses and those in the treatment group wore the SEED 1 dayPure EDOF Mid (+1.50D) contact lenses (base curve/diameter: 8.4 mm/14.2 mm) (https://www.seed.co.jp/en/products/contact/soft/1daypure_edof.html). The contact lenses were dispensed for use on a daily disposable wear schedule. This lens contains Zwitterionic material SIB) (SEED Ionic Bond) which helps to improve electrical stability and ensures high water content while keeping out dust and impurities. The design of the EDOF lens is based on the manipulation of selective higher-order spherical aberrations (as described in a patent application^22^) to achieve a thorough focus global (both central and periphery) retinal image quality that was optimised for points on and anterior to the retina, and degraded for points falling posterior to the retina to control myopia progression. The refractive profile of the lens across the optic zone was non-monotonic, non-aspheric, non-diffractive, aperiodic and refractive power profile across the optic zone diameter.^16 23^

### Procedure

At the baseline visit, participants were assessed for their eligibility, and various parameters were documented (age, age of apparent onset of myopia, number of myopic parents and information related to time spent outdoors and near work, refraction, visual acuity and ocular health status). The accommodative error (lead/lag) was determined using the monocular estimation method of dynamic retinoscopy performed at 40 cm. Later, participants were cyclopleged with 1% tropicamide (two drops, 5 min apart, regimen recommended by International Myopia Institute) preceded by topical anaesthesia (Paracain eye drops 5 mL). Thirty min after the instillation of eye-drops, the pupillary reaction was assessed with torchlight, and measurements were obtained when the pupillary response was absent. An average of five measurements was recorded from each eye using an open-field autorefractor (Shin-Nippon N Vision K 5001, Japan) to determine central and peripheral refraction up to ±30^o^ at horizontal retinal meridian based on protocols used previously.^18^ Axial length (AL) (average of three measurements) was determined using a non-contact biometer (Lenstar LS 900, Haag Streit, Switzerland). Cycloplegic SER and AL were measured at the baseline (first visit) and 12th month (final visit). Changes in SER and AL between the two groups (control and treatment) were compared after 1 year. The study followed the Consolidated Standards of Reporting Trials requirements for reporting the results.^24^

The contact lens power was appropriately adjusted based on the vertex distance calculation. Apart from the baseline and final visit of data collection, participants were asked to come to the myopia research lab at 6-month intervals to collect or replace the lenses (in case of myopia progression of ≥0.50D). For the qualitative assessment of vision and comfort related to contact lens wear as well as compliance, children were asked to fill out the questionnaire (provided as online supplemental material as eQ1) at the final visit. The compliance was self-reported in terms of the number of hours in a day that participants wore the contact lens.

10.1136/bjo-2023-323651.supp1 Supplementary data

### Outcomes

The primary outcomes were the 1-year change in spherical equivalent refractive error and axial length in the intervention and control group from the baseline. The secondary outcome variables were comfort, visual experience and compliance during the 12th month of the follow-up visit.

### Sample size

We calculated the sample size using the G*Power V.3.1.9.4 application. Based on a previous study that investigated four different designs of EDOF lenses,^14^ we obtained an average effect size ranging from 0.71 based on the mean (along with SD) change in axial length in the control group and treatment groups. Based on the level of significance of 5%, power of 90% and a one-tailed distribution, a total sample of 70 participants (35 participants per group) were required to complete a 1-year study. Adjusting for 50% of the attrition rate (on the account of COVID-19 lockdown) yielded a total sample size of 105 participants at the baseline visit.

### Statistical analysis

Statistical analysis was performed using IBM SPSS statistical software V.21 (SPSS). Graphs were created with the in-built features of Microsoft Excel 2019 (Microsoft, Albuquerque, New Mexico, USA). The Shapiro-Wilk test for normality indicated that the data were normally distributed (p>0.05), and therefore, parametric tests were performed to check the statistical significance. Independent t-tests were applied for the comparison of parameters between the treatment and control groups. Paired t-tests were applied to test the significance of relative peripheral refraction between the baseline and 12-month visits in both control and treatment groups. The Pearson correlation coefficient was used to determine the relationship between the two parameters in this study. The factors associated with myopia progression and axial elongation such as age, gender, age of apparent onset of myopia, baseline myopia, myopic parents, and time spent outdoors and near work were analysed using a multiple linear regression model. Intention-to-treat (ITT) analysis was also performed for the participants who lost to follow-up to test the efficacy of the treatment lens.

In this study, we defined ‘responders’ for the treatment as the eyes in the treatment group that progressed less than the mean changes of eyes in the control group, and ‘non or poor-responders’ for the treatment were defined as the eyes in the treatment group that progressed the same or showed greater progression compared with the control eyes. In the control group, we defined the non-progressors as those with no change in SER and axial length (AL) in the 12th-month visit compared with the baseline visit. For all tests, the criteria for the statistical significance were set as p<0.05.

## Results

### Participants profile

**Figure 1:**
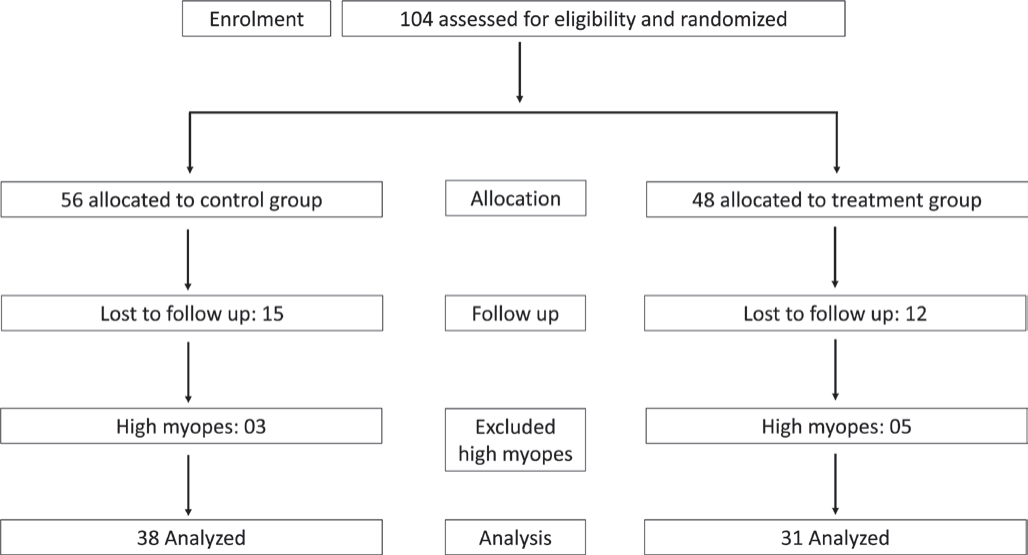
Flow chart of the study.

Figure 1 shows the flow chart of the number of participants recruited, enrolled and dropped out of the study. One hundred and four eligible participants were enrolled and randomised to wear either single-vision spectacles (n=56) or treatment contact lenses (n=48). Of the 104 participants, 27 discontinued before completing the study (control: 27% (15/56) and treatment: 25% (12/48)). The reasons for discontinuation were as follows: no longer interested in wearing contact lenses (03/27), lens handling issues (02/27) and not interested in attending follow-up due to COVID-19 or migration (22/27). High myopia (SER≤−6.00D) was noted in 12 participants at baseline (6 in each group) and 8 participants who completed 12-month follow-up (5 in the treatment group and 3 in the control group) who were excluded due to a smaller sample size. Further analysis was conducted in participants with low myopia (≤−0.50D to >−6.00D, ie, 92 participants at baseline and 69 participants who completed 12-month follow-up. There was no difference in the baseline characteristics of the participants who completed the 12-month follow-up and those who were lost to follow-up (online supplemental table S1).

### Baseline measures

Table 1 shows the baseline characteristics of all participants with low myopia who were randomly allocated to the treatment and control groups. In brief, the mean±SD age of participants recruited (n=92) in this study was 11.31±2.58 years, the mean spherical equivalent refractive error was −3.32±1.43D and the mean axial length was 24.42±0.75 mm. There were no statistical differences (p>0.05) in the baseline characteristics between the treatment and the control group in those who were enrolled, and those who completed 12 months visit (table 1). While the difference of baseline SER obtained through cycloplegic autorefraction between the control and treatment group was not statistically significant, it is worth highlighting that there was a marginal difference between the groups by 0.55D that is of clinical relevance.

**Table 1:** Baseline data of all participants recruited and the participants who completed the 12-month visit

All enrolled participants (n=92)	Baseline data, mean±SD		
	Control (n=50)	Treatment (n=42)	P value
Age at recruitment (years)	11.06±2.45	11.31±2.58	0.64
Age of myopia onset (years)	8.94±2.50	8.81±2.42	0.80
Gender (%, male:female)	56:44	45:55	0.30
No of myopic parents (none:1:2)	26:17:07	14:19:09	0.19
Cycloplegic autorefraction in SER (D)	−2.77±1.32	−3.32±1.43	0.06
Axial length (mm)	24.24±0.91	24.42±0.75	0.30
Accommodative lag at 40 cm (D)	0.42±0.36	0.46±0.28	0.55

Participants completed 12-month visit (n=69)	Control (n=38)	Treatment (n=31)	P value
Age at recruitment (years)	11.03±2.69	11.26±2.54	0.71
Age of myopia onset (years)	9.37±2.62	8.61±2.22	0.20
Gender (%, male:female)	55:45	42:58	0.27
No of myopic parents (none:1:2)	18:15:05	10:13:08	0.30
Cycloplegic autorefraction in SER (D)	−2.69±1.27	−3.28±1.44	0.08
Axial length (mm)	24.16±0.90	24.40±0.80	0.25
Accommodative lag at 40 cm (D)	0.41±0.39	0.44±0.30	0.73

%, percentage; D, dioptre; mm, millimetres; n, number of participants; SER, spherical equivalent refraction.

### Change in SER and axial length

**Figure 2:**
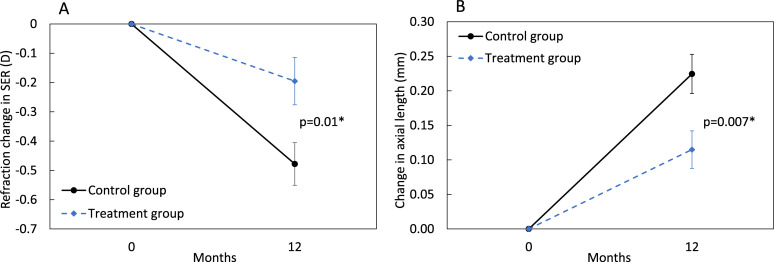
The mean change in spherical equivalent refraction (A: left panel) and axial length (B: right panel) between the baseline and 12-month period. Error bars represent the SE of the mean. p<0.05, indicated by asterisks*.

For the 69 participants who visited and completed the 12-month follow-up, the mean±SEM change in SER for the control and treatment group was −0.48±0.07D and −0.20±0.08D, respectively (figure 2A). Corresponding mean changes in AL for control and treatment groups were 0.22±0.03 mm and 0.11±0.03 mm, respectively (figure 2B). The participants in the treatment group showed significantly smaller changes in SER and AL than that of the control group (SER: mean difference=−0.28D, p=0.01, 59%; AL: mean difference=0.11 mm, p=0.007, 49%). Pearson correlation indicated a negative significant correlation between the changes in AL and the changes in SER in both controls (r=−0.55, p<0.001) and for the treatment group (r=−0.55, p=0.01). In a separate analysis, those (41 participants) who completed all the follow-up visits based on the initial protocol, the mean±SEM change in the SER for the control versus treatment group was −0.11±0.10D vs 0.11±0.21D at 3 months, −0.34±0.15D vs −0.10±0.07D at 6 months and −0.59±0.13D vs −0.20±0.09D at 12 months (online supplemental figure S1); corresponding changes in AL were 0.08±0.02 mm vs 0.04±0.02 mm, 0.16±0.03 mm vs 0.07±0.02 mm and 0.28±0.04 mm vs 0.11±0.03 mm, respectively.

In the ITT analysis that included all 92 participants, the adjusted mean changes in myopia progression were −0.49±0.06D in the control group and −0.18±0.06D in the treatment group. The corresponding mean axial length elongation for control and treatment groups after the adjustment (lost to follow-up) were 0.23±0.02 mm and 0.12±0.02 mm, respectively. The participants who wore treatment lenses had significantly less myopia progression by 63% (mean difference: −0.31D, p=0.001) and less axial elongation by 48% (mean difference: 0.11 mm, p=0.001).

For this study, multiple linear regression analysis indicated that the age, age of apparent onset of myopia, number of myopic parents, baseline myopia, gender, time spent outdoors and time spent on near work activities did not influence the changes in SER (F _(7,61)_ 0.72, p=0.66, R^2^=0.08) and AL (F _(7,61)_ 2.00, p=0.07, R^2^=0.19).

### Responders versus non-responders

In the treatment group, 74% (23/31) and 77% (24/31) of participants responded to the treatment (EDOF lenses) showing lesser changes in SER and AL, respectively, than in the control group. There were 42% (13/31) and 19% (6/31) of participants did not undergo any changes in SER and AL, respectively, in the treatment group. In contrast, only 16% (6/38) and 11% (4/38) of the children in the control group did not undergo change in SER and AL, respectively.

### Subjective responses from the participants who wore contact lenses in the treatment group

**Figure 3:**
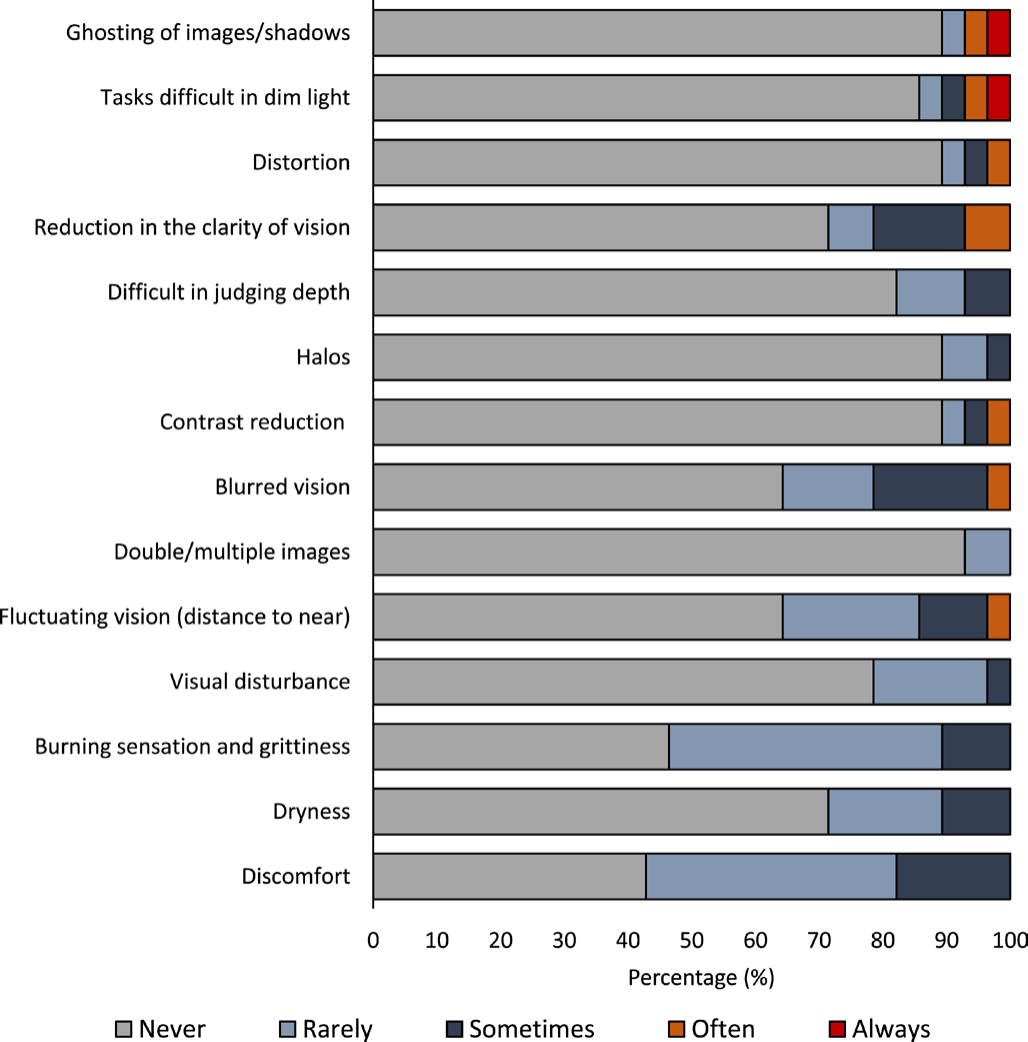
Percentage responses from the participants related to comfort, visual disturbances and compliance for those who wore the contact lenses in the treatment group.

Participants in the treatment group wore their contact lenses in both eyes for an average time (mean±SD) of 12.0±2.67 hours/day. The number of days the participants wore the contact lens is not quantified, though all of them verbally reported wearing all the days during the trial. None of the participants reported adverse effects with the contact lenses. Figure 3 shows the subjective response of participants related to discomfort and visual disturbances for those in the treatment group. While the majority of the participants (82%, n=23/28) were comfortable with the contact lenses, 18% (n=5/28) reported discomfort, 11% (n=3/28) reported occasional dryness (sometimes) and 14% (n=4/28) reported mild fluctuations in visual acuity after immediate lens wear (sometimes or often) which subsided later.

## Discussion

This 1-year randomised clinical trial study had three findings. First, the treatment lenses showed a significant reduction in myopia progression and axial length elongation compared with that of the conventional single-vision spectacles. Second, all the participants adapted to the contact lenses and did not report any major adverse effects. Third, participants’ current age, age of apparent onset of myopia, gender, number of myopic parents, baseline myopia, time spent outdoors and time spent near work did not influence the treatment effect.

In the current study, we found that axial elongation was slowed down by 0.11 mm (49%) with 1 year of use of the EDOF design lens (+1.50D). The outcomes from the current study agree with findings from multiple studies that investigated the efficacy of peripheral defocus myopia control lenses which were designed to counteract relative peripheral hyperopia (table 2).^13^ Ruiz-Pomeda *et al* reported a similar treatment efficacy of 59% (0.12 mm) with MiSight contact lenses in the Spanish cohort in comparison to the present study within a year.^25^ In a cross-over study for 10 months, Anstice and Phillips^26^ reported 50% (0.13 mm/year) retardation of axial elongation with the dual focus contact lens (2D myopic defocus) compared with that of the single vision lenses. Recently, Weng *et al* reported a similar efficacy for controlling myopia progression with both EDOF and MiSight (centre-distance dual focus) contact lenses.^15^ Similarly, Sankaridurg *et al* reported slowing of myopia progression by 30% (−0.20 D) and axial elongation by 33% (0.11 mm) with the EDOF test lens III (+1.75D), while the corresponding reduction in myopia progression and axial elongation with the EDOF test lens IV (+1.25D) was 26% (−0.17 D) and 23% (0.11 mm) when conventional single vision contact lenses were used as control.^14^ The present study also showed similar test efficacy for change in SER and axial length per year with EDOF lenses (+1.50D) compared with the EDOF lenses (+1.75D and+1.25D) used by Sankaridurg *et al* (change in SER: −0.28 D vs −0.20 D and −0.17 D; change in AL: 0.11 mm vs 0.11 mm). Based on the cumulative absolute reduction in axial elongation, the outcomes among ethnicities seem similar and the efficacy of myopia control appears to be independent of ethnicity and benefit remains the same with any myopia control modalities as indicated by Bullimore and Brennan,^27^ provided the refractive error and age are comparable as the severity of myopia and age of individuals affects the rate of myopia progression.^28^

**Table 2:** Summary of previous clinical studies of defocus lenses to control myopia progression

Authors (years)	Duration (months)	Study design	Age (years)	Ethnicity	Entry Rx range (D)	Intervention (control, treatment)	Sample size (n)	SER progression per year		Axial elongation per year	
								Absolute change (mm)	Percentage change (%)	Absolute change (mm)	Percentage change (%)
Lam *et al* 11 (2013)	24	Randomised, masked	8–13	Chinese	−1.00 to −5.00	SV CL DISC	47 49	−0.12	25	0.08	38
Anstice and Philips^26^ (2011)	10	Randomised, cross over	11–14	Multiethnic	−1.25 to −4.50	SV CL DF	40 40	−0.30	37	0.13	50
Sankaridurg *et al* 13 (2011)	12	Randomised	7–14	Chinese	−0.75 to −3.50	SV SP Novel CL	40 45	−0.29	34	0.13	33
Sankaridurg *et al* 14 (2019)	24	Randomised, double-masked	7–13	Chinese	−0.75 to −3.50	SV CL EDOF CL 1 EDOF CL 2 EDOF CL 3 EDOF CL 4	102 103 101 98 104	−0.20 −0.14 −0.20 −0.17	30 21 30 26	0.14 0.10 0.11 0.11	42 30 33 33
Lam *et al* 10 (2019)	24	Randomised, double-masked	8–13	Hong Kong Chinese	−1.00 to −5.00	SV SP DIMS SP	81 79	−0.38	69	0.21	66
Bao *et al* 30 (2021)	12	Randomised, double-masked	8–13	Chinese	−0.75 to −4.75	SV SP HAL SP SAL SP	52 54 55	−0.53 −0.33	67 41	0.23 0.11	64 31
Chamberlain *et al* 12 (2019)	36	Randomised, double-masked	8–12	Multiethnic	−0.75 to −4.00	SV CL DF CL	74 70	−0.40	69	0.15	63
Walline *et al* 31 (2013)	24	Matched study group	8–11	White	−1.00 to −6.00	SV CL MF CL	32 32	−0.27	45	0.07	32
Ruiz-Pomeda *et al* 25 (2018)	24	Randomised, masked	8–12	Spanish	−0.75 to −4.00	SV SP DF CL	33 41	−0.26	59	0.12	50
Present study	12	Randomised	7–15	Indian	−0.50 to −5.75	SV SP EDOF CL	38 31	−0.28	59	0.11	49

CL, contact lens; DF, dual focus; DIMS, defocus incorporated multiple segmented; DISC, defocus incorporated soft contact lens; EDOF, extended depth of focus; HAL, highly aspherical lenslet; MF, multifocal; SAL, slightly aspherical lenslet; SER, spherical equivalent refraction; SP, spectacles; SV, single vision.

We found that there was a myopic shift in the relative peripheral refraction at the horizontal retinal eccentricities during the final visit (12th month) compared with the baseline visit in the treatment group (online supplemental figure S2). Given that the relative peripheral refraction is considered to be associated with retinal shape,^29^ based on our findings related to changes in relative peripheral refraction, we speculate that EDOF lenses may have an influence in altering the retinal shape during the treatment period. However, future studies are required to understand the exact mechanism of how the EDOF contact lenses or defocus lenses influence the retinal shape to control the progression of myopia and axial elongation.

In the present study, participants wore contact lenses for an average of 8–12 hours per day, and the treatment effect with the SEED 1 dayPure EDOF Mid contact lens was consistent with the study findings by Lam *et al*.^11^ They reported that the treatment effect of controlling myopia progression increased from 46% to 58% when the participants wore the contact lenses (DISC) for 7 hours or more per day.^11^ However, further studies are needed to understand the minimum wearing hours per day to attain maximum treatment effect with EDOF lenses to control myopia progression.

This study has a few limitations. First, the use of single-vision spectacles in the control participants might not be appropriate which resulted in a lack of masking for the participants. Second, one-fourth (25%) of participants were lost to follow-up at the final visit. We noted that there was migration of participants during the COVID-19 lockdown in India and this could be one of the reasons. Third, we did not measure the pupil size of participants to understand how pupillary diameter influences the efficacy of test lenses to control the progression of myopia. Fourth, due to the inadequate sample size to compare the control and treatment groups, we did not include individuals with high myopia, and the findings are limited to low myopes (≤−0.50D to>−6.00D). We recommended future studies to include high myopes (<−6.00D) to test the efficacy of EDOF lenses in controlling the progression of myopia

## Conclusion

In summary, our results demonstrated that daily wear of SEED 1 dayPure EDOF Mid (+1.50 D) contact lens was effective in controlling myopia progression and axial elongation compared with conventional single-vision spectacles in Indian children. No adverse effects were reported with the contact lenses ensuring the safety and comfort of lens usage in children for myopia control.

## Data Availability

Data are available on reasonable request.

## References

[1] Pan CW , Ramamurthy D , Saw SM . Worldwide prevalence and risk factors for myopia. Ophthalmic Physiol Opt 2012;32:3–16. 10.1111/j.1475-1313.2011.00884.x 22150586

[2] Holden BA , Fricke TR , Wilson DA , et al . Global prevalence of myopia and high myopia and temporal trends from 2000 through 2050. Ophthalmology 2016;123:1036–42. 10.1016/j.ophtha.2016.01.006 26875007

[3] Lawrenson JG , Shah R , Huntjens B , et al . Interventions for myopia control in children: a living systematic review and network meta-analysis. Cochrane Database Syst Rev 2023;2:CD014758. 10.1002/14651858.CD014758.pub2 36809645 PMC9933422

[4] Bakaraju RC , Ehrmann K , Papas EB , et al . Do peripheral refraction and aberration profiles vary with the type of mmyopia? - An illustration using a ray-tracing approach. J Optom 2009;2:29–38. 10.3921/joptom.2009.29

[5] Tabernero J , Vazquez D , Seidemann A , et al . Effects of myopic spectacle correction and radial refractive gradient spectacles on peripheral refraction. Vision Res 2009;49:2176–86. 10.1016/j.visres.2009.06.008 19527743

[6] Mutti DO , Sholtz RI , Friedman NE , et al . Peripheral refraction and ocular shape in children. Invest Ophthalmol Vis Sci 2000;41:1022–30. 10752937

[7] Seidemann A , Schaeffel F , Guirao A , et al . Peripheral refractive errors in myopic, emmetropic, and hyperopic young subjects. J Opt Soc Am A Opt Image Sci Vis 2002;19:2363–73. 10.1364/josaa.19.002363 12469730

[8] Mutti DO , Sinnott LT , Mitchell GL , et al . Relative peripheral refractive error and the risk of onset and progression of myopia in children. Invest Ophthalmol Vis Sci 2011;52:199–205. 10.1167/iovs.09-4826 20739476 PMC3053275

[9] Sankaridurg P , Donovan L , Varnas S , et al . Spectacle lenses designed to reduce progression of myopia: 12-month results. Optom Vis Sci 2010;87:631–41. 10.1097/OPX.0b013e3181ea19c7 20622703 PMC4696394

[10] Lam CSY , Tang WC , Tse DY-Y , et al . Defocus incorporated multiple segments (DIMS) spectacle lenses slow myopia progression: a 2-year randomised clinical trial. Br J Ophthalmol 2020;104:363–8. 10.1136/bjophthalmol-2018-313739 31142465 PMC7041503

[11] Lam CSY , Tang WC , Tse DY-Y , et al . Defocus incorporated soft contact (DISC) lens slows myopia progression in Hong Kong Chinese schoolchildren: a 2-year randomised clinical trial. Br J Ophthalmol 2014;98:40–5. 10.1136/bjophthalmol-2013-303914 24169657 PMC3888618

[12] Chamberlain P , Peixoto-de-Matos SC , Logan NS , et al . A 3-year randomized clinical trial of MiSight lenses for myopia control. Optom Vis Sci 2019;96:556–67. 10.1097/OPX.0000000000001410 31343513

[13] Sankaridurg P , Holden B , Smith E , et al . Decrease in rate of myopia progression with a contact lens designed to reduce relative peripheral hyperopia: one-year results. Invest Ophthalmol Vis Sci 2011;52:9362–7. 10.1167/iovs.11-7260 22039230

[14] Sankaridurg P , Bakaraju RC , Naduvilath T , et al . Myopia control with novel central and peripheral plus contact lenses and extended depth of focus contact lenses: 2 year results from a randomised clinical trial. Ophthalmic Physiol Opt 2019;39:294–307. 10.1111/opo.12621 31180155 PMC6851825

[15] Weng R , Lan W , Bakaraju R , et al . Efficacy of contact lenses for myopia control: insights from a randomised, contralateral study design. Ophthalmic Physiol Opt 2022;42:1253–63. 10.1111/opo.13042 36006761 PMC9805073

[16] Bakaraju RC , Ehrmann K , Ho A . Extended depth of focus contact lenses vs. two commercial multifocals: part 1. Optical performance evaluation via computed through-focus retinal image quality metrics. J Optom 2018;11:10–20. 10.1016/j.optom.2017.04.003 28606456 PMC5777930

[17] Bakaraju RC , Tilia D , Sha J , et al . Extended depth of focus contact lenses vs. two commercial multifocals: part 2. Visual performance after 1 week of lens wear. J Optom 2018;11:21–32. 10.1016/j.optom.2017.04.001 28619486 PMC5777928

[18] Yelagondula VK , Achanta DSR , Panigrahi S , et al . Asymmetric peripheral refraction profile in myopes along the horizontal meridian. Optom Vis Sci 2022;99:350–7. 10.1097/OPX.0000000000001890 35383734

[19] Chen X , Sankaridurg P , Donovan L , et al . Characteristics of peripheral refractive errors of myopic and non-myopic Chinese eyes. Vision Res 2010;50:31–5. 10.1016/j.visres.2009.10.004 19825388

[20] Kang P , Gifford P , McNamara P , et al . Peripheral refraction in different ethnicities. Invest Ophthalmol Vis Sci 2010;51:6059–65. 10.1167/iovs.09-4747 20505193

[21] Atchison DA , Pritchard N , Schmid KL . Peripheral refraction along the horizontal and vertical visual fields in myopia. Vision Res 2006;46:1450–8. 10.1016/j.visres.2005.10.023 16356528

[22] Bakaraju RC , Ehrmann K , Ho A , et al . Inventors lenses, devices, methods and systems for refractive error. Google patents. 2014.

[23] Tilia D , Bakaraju RC , Chung J , et al . Short-term visual performance of novel extended depth-of-focus contact lenses. Optom Vis Sci 2016;93:435–44. 10.1097/OPX.0000000000000806 26808384

[24] Schulz KF , Altman DG , Moher D , et al . CONSORT 2010 statement: updated guidelines for reporting parallel group randomised trials. BMC Med 2010;8:18. 10.1186/1741-7015-8-18 20334633 PMC2860339

[25] Ruiz-Pomeda A , Pérez-Sánchez B , Valls I , et al . Misight assessment study Spain (MASS). A 2-year randomized clinical trial. Graefes Arch Clin Exp Ophthalmol 2018;256:1011–21. 10.1007/s00417-018-3906-z 29396662

[26] Anstice NS , Phillips JR . Effect of dual-focus soft contact lens wear on axial myopia progression in children. Ophthalmology 2011;118:1152–61. 10.1016/j.ophtha.2010.10.035 21276616

[27] Bullimore MA , Brennan NA . Efficacy in myopia control: does race matter?. Optom Vis Sci 2023;100:5–8. 10.1097/OPX.0000000000001977 36705709

[28] Verkicharla PK , Kammari P , Das AV . Myopia progression varies with age and severity of myopia. PLoS One 2020;15:e0241759. 10.1371/journal.pone.0241759 33216753 PMC7678965

[29] Verkicharla PK , Suheimat M , Schmid KL , et al . Peripheral refraction, peripheral eye length, and retinal shape in myopia. Optom Vis Sci 2016;93:1072–8. 10.1097/OPX.0000000000000905 27281680

[30] Bao J , Yang A , Huang Y , et al . One-year myopia control efficacy of spectacle lenses with aspherical lenslets. Br J Ophthalmol 2022;106:1171–6. 10.1136/bjophthalmol-2020-318367 33811039 PMC9340037

[31] Walline JJ , Greiner KL , McVey ME , et al . Multifocal contact lens myopia control. Optom Vis Sci 2013;90:1207–14. 10.1097/OPX.0000000000000036 24061152

