# Supplementary material for: Randomised clinical trial of extended depth of focus lenses for controlling myopia progression: Outcomes from SEED LVPEI Indian Myopia Study
Source: Br J Ophthalmol. 2024 Apr 11;108(9):1292–8. doi: 10.1136/bjo-2023-323651 (PMC11347265; doi:10.1136/bjo-2023-323651)
Supplement: Supplementary data [file bjo-2023-323651supp001.pdf]

Supplementary content

Title: Randomised Clinical Trial of extended depth of focus lenses for controlling myopia progression - Outcomes from SEED LVPEI Indian Myopia Study (SLIMS)

Authors:

Manoj K. Manoharan and Pavan K. Verkicharla

eQ1: Questionnaire

1. How many hours did you wear the contact lenses during the day?

    a) Less than or equal to 1 hour

    b) Less than or equal to 4 hours

    c) Less than or equal to 8 hours

    d) Less than or equal to 12 hours

    e) Less than or equal to 16 hours

2. Did your eyes feel discomfort while wearing contact lenses?

    a) Never b) Rarely c) Sometimes d) Often e) Always

3. Did you experience a feeling of dryness to your eyes with your contact lens?

    a) Never b) Rarely c) Sometimes d) Often e) Always

4. Did you experience the burning sensation, grittiness (feels like sand in the eye), and awareness of the contact lenses in your eyes?

    a) Never b) Rarely c) Sometimes d) Often e) Always

5. Did your eyes bother you to remove the contact lenses/spectacles while doing a task, when you experienced the visual disturbance?

    a) Never b) Rarely c) Sometimes d) Often e) Always

6. Did you experience that your vision was fluctuating (blur) when you changed focus from distance to near with your contact lenses?

    a) Never b) Rarely c) Sometimes d) Often e) Always

7. Did you experience double images or multiple images with your contact lenses while viewing either distance, intermediate or near target/work?

    a) Never b) Rarely c) Sometimes d) Often e) Always

8. Did you experience blurred vision when looking far away objects with your contact lenses (E.g.; watching television, mountains, and buildings in the distance)?

   a) Never b) Rarely c) Sometimes d) Often e) Always

9. Did you experience the reduction in contrast of the object during the task with your contact lenses?

   a) Never b) Rarely c) Sometimes d) Often e) Always

10. Did you experience halos (ring like shadow around the light) with your contact lenses during the wearing hours?

    a) Never b) Rarely c) Sometimes d) Often e) Always

11. Did you experience difficulty in judging depth or distance of the target from your eye with your contact lenses?

    a) Never b) Rarely c) Sometimes d) Often e) Always

12. Did you notice the reduction in the clarity of vision with your contact lenses during distance, intermediate or near viewing?

    a) Never b) Rarely c) Sometimes d) Often e) Always

13. Did you experience distortion (or waviness) with your contact lenses while seeing the objects?

    a) Never b) Rarely c) Sometimes d) Often e) Always

14. Did you experience difficulty during doing tasks in dim light with your contact lenses?

    a) Never b) Rarely c) Sometimes d) Often e) Always

15. Did you experience ghosting of images/ shadows with your contact lenses for distance, intermediate, and near?

    a) Never b) Rarely c) Sometimes d) Often e) Always

Supplementary table S1

Supplementary Table S1: *Baseline data of all participants lost to follow-up at 12-month visit and the participants who completed the 12-month visit.*

	Baseline data, Mean ± SD		
	Completed	Lost to follow up	*p* Value
**All enrolled participants (n = 92)**	n = 69	n = 23	
Age at recruitment (years)	11.2 ± 2.5	11.0 ± 2.5	1.00
Age of myopia onset (years)	9.0 ± 2.5	8.4 ± 2.4	0.33
Gender (%, Male: Female)	49: 51	57: 43	0.55
Number of myopic parents (None: 1: 2)	41: 40: 19	42: 35: 13	0.60
Cycloplegic autorefraction in SER (D)	-3.01 ± 1.32	-3.21 ± 1.52	0.18
Axial length (mm)	24.29 ± 0.87	24.49 ± 0.78	0.60
Accommodative lag at 40 cm (D)	0.42 ± 0.01	0.48 ± 0.01	0.97
**Controls participants (n = 50)**	n = 38	n = 12	
Age at recruitment (years)	11.1 ± 2.5	10.9 ± 2.2	0.50
Age of myopia onset (years)	9.4 ± 2.6	7.6 ± 1.4	0.12
Gender (%, Male: Female)	55: 45	58: 42	0.85
Number of myopic parents (None: 1: 2)	47: 39: 13	67: 17: 17	0.35
Cycloplegic autorefraction in SER (D)	-2.79 ± 1.40	-2.95 ± 1.58	0.37
Axial length (mm)	24.20 ± 0.91	24.48 ± 0.93	0.87
Accommodative lag at 40 cm (D)	0.40 ± 0.39	0.43 ± 0.20	0.67
**Treated participants (n = 42)**	n = 31	n = 11	
Age at recruitment (years)	11.3 ± 2.5	11.5 ± 2.8	0.58
Age of myopia onset (years)	8.6 ± 2.2	9.4 ± 3.0	0.20
Gender (%, Male: Female)	42: 58	55: 45	0.47
Number of myopic parents (None: 1: 2)	32: 42: 26	36: 55: 9	0.50
Cycloplegic autorefraction in SER (D)	-3.27 ± 1.38	-3.49 ± 1.48	0.61
Axial length (mm)	24.39 ± 0.76	24.49 ± 0.62	0.55
Accommodative lag at 40 cm (D)	0.45 ± 0.27	0.53 ± 0.18	0.96

n = number of participants, D = dioptre, and mm = millimetres

Supplementary figure S1

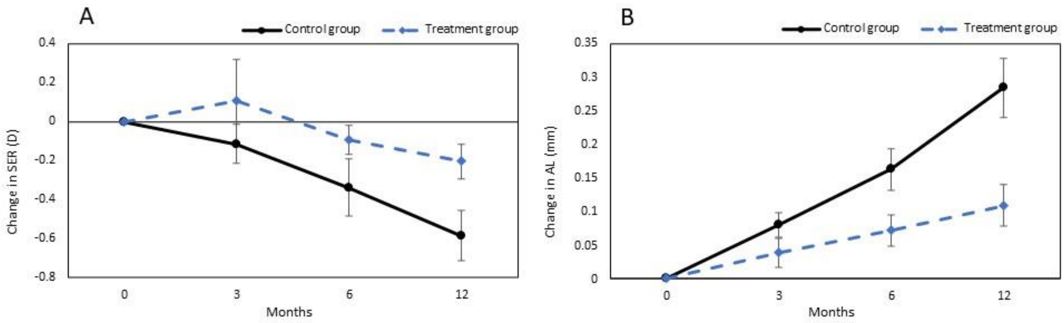

Supplementary Figure 1: *The mean change in spherical equivalent refraction (left panel) and axial length (right panel) from the baseline to 3-, 6-, and 12-months period. Error bars represent the standard error of the mean.*

Supplementary Figure S2

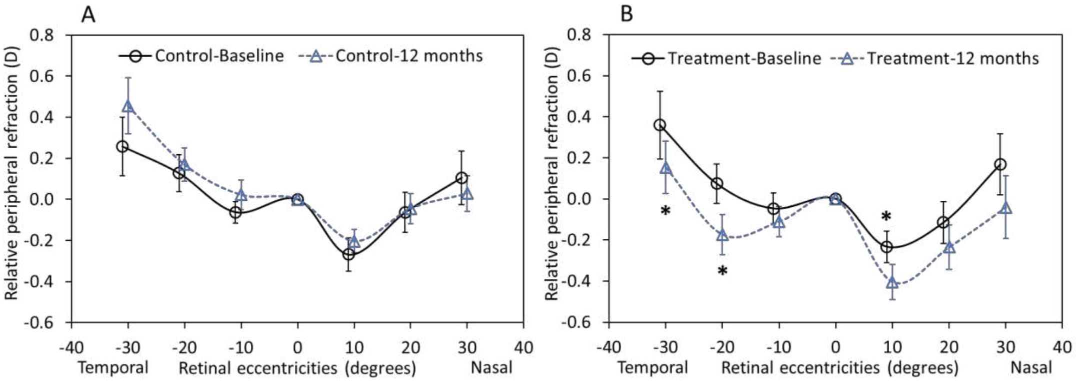

Supplementary Figure S2: *Relative peripheral refraction plotted as the function of retinal eccentricity for baseline and 12 months of control and treatment groups at horizontal retinal meridian. Error bars indicate the standard error of the mean. The figure indicates that in the treatment group, the relative peripheral refraction pattern at 12 months visit had a significant shift towards myopic direction compared to the baseline visit at temporal 20°, temporal 30°, and nasal 10° retina ($p<0.05$, indicated by asterisks*).*
